# An automated and high-throughput-screening compatible pluripotent stem cell-based test platform for developmental and reproductive toxicity assessment of small molecule compounds

**DOI:** 10.1007/s10565-020-09538-0

**Published:** 2020-06-20

**Authors:** Gesa Witt, Oliver Keminer, Jennifer Leu, Rashmi Tandon, Ina Meiser, Anne Willing, Ingo Winschel, Jana-Christin Abt, Björn Brändl, Isabelle Sébastien, Manuel A. Friese, Franz-Josef Müller, Julia C. Neubauer, Carsten Claussen, Heiko Zimmermann, Philip Gribbon, Ole Pless

**Affiliations:** 1 Fraunhofer IME ScreeningPort, Schnackenburgallee 114, 22525 Hamburg, Germany; 2 Fraunhofer IBMT, 66280 Sulzbach, Saar Germany; 3 Institut für Neuroimmunologie und Multiple Sklerose, Zentrum für Molekulare Neurobiologie Hamburg, Universitätsklinikum Hamburg-Eppendorf, 20251 Hamburg, Germany; 4 Christian-Albrechts-Universität zu Kiel, ZIP gGmbH, 24105 Kiel, Germany; 5 Lehrstuhl für Molekulare und Zelluläre Biotechnologie, Universität des Saarlandes, 66123 Saarbrücken, Germany; 6 Fakultät für Meereswissenschaften, Universidad Católica del Norte, CL-1781421 Coquimbo, Chile

**Keywords:** Developmental toxicity, Embryonic stem cells, Induced pluripotent stem cells, High-throughput, Flow cytometry, Automation

## Abstract

The embryonic stem cell test (EST) represents the only validated and accepted in vitro system for the detection and classification of compounds according to their developmental and reproductive teratogenic potency. The widespread implementation of the EST, however, in particular for routine application in pharmaceutical development, has not been achieved so far. Several drawbacks still limit the high-throughput screening of potential drug candidates in this format: The long assay period, the use of non-homogeneous viability assays, the low throughput analysis of marker protein expression and the compatibility of the assay procedures to automation. We have therefore introduced several advancements into the EST workflow: A reduction of the assay period, an introduction of homogeneous viability assays, and a straightforward analysis of marker proteins by flow cytometry and high content imaging to assess the impact of small molecules on differentiation capacity. Most importantly, essential parts of the assay procedure have been adapted to lab automation in 96-well format, thus enabling the interrogation of several compounds in parallel. In addition, extensive investigations were performed to explore the predictive capacity of this next-generation EST, by testing a set of well-known embryotoxicants that encompasses the full range of chemical-inherent embryotoxic potencies possible. Due to these significant improvements, the augmented workflow provides a basis for a sensitive, more rapid, and reproducible high throughput screening compatible platform to predict in vivo developmental toxicity from in vitro data which paves the road towards application in an industrial setting.

**Electronic supplementary material:**

The online version of this article (10.1007/s10565-020-09538-0) contains supplementary material, which is available to authorized users.

**Graphical abstract:**
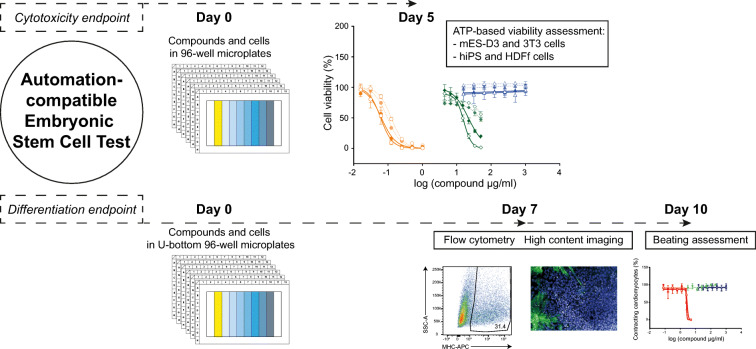
•The embryonic stem cell test to predict teratogenicity was made automation-compatible. •Several key improvements to the assay procedure have been introduced to increase performance. •The workflow was adapted to human iPS cells and isogenic fibroblast donor cells.

## Introduction

In drug discovery, it is mandatory to evaluate drug side effects in early preclinical stages of compound development, in particular, those that impact the physiological development of the fetus and can lead to growth retardation or teratogenesis. The vast number of tissue targets for exogenic induction of malformations during embryonic development is the rationale for toxicity testing of chemicals in highly standardized animal experiments according to Organization for Economic Co-operation and Development (OECD) test guidelines. These guidelines generally specify time-consuming and expensive in vivo experiments mostly performed with mammalian species such as rats or rabbits. For both ethical and economical reasons, there is therefore a great demand for alternatives to the testing of chemical-induced adverse effects on reproduction and development in living mammals.

More than 20 years ago, an in vitro model to assess embryotoxicity in mouse cells was proposed (Spielmann et al. 1997) and has since been widely applied, extended (Seiler et al. 2004), and validated (Genschow et al. 2004; Scholz et al. 1999). This embryonic stem cell test (EST) is based on the assessment of three toxicological endpoints after several days of chemical exposure, namely (1) the cytotoxic effect on a mouse embryonic stem cell (mESC) line (D3) and on (2) mouse NIH/3T3 fibroblasts and (3) the morphological analysis of beating in mESC-derived cardiomyocytes. One of the best-studied models of mESC differentiation is the formation of multicellular aggregates called embryoid bodies (EBs) in suspension culture (Martin et al. 1977). Within these aggregates, complex interactions between heterologous cell types result in the induction of differentiation of stem cells to derivatives of all three embryonic germ layers (Doetschman et al. 1985). In addition, it takes advantage of the potential of mESCs to differentiate into a variety of cell types upon withdrawal of leukemia inhibitory factor (LIF). Plating of the EBs on extracellular matrix substituents allows further differentiation and EB outgrowth. In the presence of FBS, the cardiomyocyte lineage is the predominant differentiation path in D3 cells, resulting in beating areas within the outgrowth. These beating areas can be assessed morphologically by microscopic analysis and scored. Over the last decade, numerous technological advances were achieved in the field of stem cell toxicology (summarized in (Luz and Tokar 2018)) utilizing mouse and human pluripotent stem cells (PSCs) and various read-outs based on metabolomics (Palmer et al. 2013), transcriptomic (Suzuki et al. 2011a), transgenic (Le Coz et al. 2015), and high-throughput imaging (Kameoka et al. 2014). In particular, alternative EST formats which are based on the assessment of compound’s impact on the expression of mesodermal reporter genes (Hand1-EST, Cyma1-EST) have been proposed (Le Coz et al. 2015; Nagahori et al. 2016; Suzuki et al. 2011b) and extensively validated (Suzuki et al. 2012). PSCs are an ideal in vitro model to investigate developmental toxicity as they possess the capacity to differentiate into specialized cells of different germ layers such as the heart, liver, or cells of the central nervous system. Therefore, they exhibit obvious advantages over non-human cells, immortalized, and primary cells. Even though EST has been implemented with human embryonic stem cells, in particular with the cell line WA09 (Kleinstreuer et al. 2011; Palmer et al. 2013; West et al. 2010), the implementation of hiPSC-based high-throughput (HT) compatible assays has not yet been achieved.

For the assessment of cytotoxicity, a variety of microtitre plate compatible assays based on colorimetric, fluorometric, and luminescence detection technologies are available. The absorbance-based methods have historically been the most widely utilized, in particular the quantification of mitochondrial succinate dehydrogenase activity of cells using the tetrazolium reagent MTT. The MTT assay, however, has been reported to be toxic to eukaryotic cells and adding the reagent to estimate cell viability may actually be damaging or even killing cells during the course of an experiment (Riss et al. 2004). Other formats, which probe the intracellular ATP concentration using a luciferase reaction, are non-toxic, show a good positive correlation between cell number and ATP concentration and are more sensitive than MTT assays (Petty et al. 1995). With regard to toxicity profiles, MTT and ATP assays generate similar pIC_50_ values for cytostatic agents like cisplatin, docetaxel, doxorubicin, or vinblastine (Mueller et al. 2004). We have therefore adapted the EST viability assessment to non-toxic homogeneous “mix-and-read” procedures based on ATP assessment, not on NADH production. The rate of tetrazolium reduction reflects the general metabolic activity or the rate of glycolytic NADH production in MTT assays. Therefore, pluripotent cells growing in monolayer or 3D configuration will have a different rate of metabolism than those that have undergone differentiation, grown into a confluent monolayer, or have become senescent. The most reliable and widely used alternative to the MTT assay is the ATP assay, which measures ATP as a surrogate marker for viability. ATP-based assays are known to be a simple, fast, and very sensitive method for measuring viable cells using a plate reader. Whereas the MTT assay requires incubation of the tetrazolium substrate with viable cells for hours to generate a color signal (followed by a second procedural step to solubilize the formazan crystals), the ATP assay reagent immediately lyses cells upon addition and generates a stable luminescent signal following a 10-min equilibration period. The ATP assay shows a typical sensitivity that is two orders of magnitude better than the MTT assay.

In this study, to establish the reliability and feasibility of the EST for industrial application during compound characterization for drug development, several optimizations to the established and validated EST protocol (Seiler and Spielmann 2011) were introduced to (1) shorten the cytotoxicity assessment procedure; (2) utilize state-of-the-art homogeneous assay formats for viability assessment based on ATP; (3) enable automation-compatibility of the workflow, in particular for cell seeding, compound dilution, media exchange, and viability assessment; (4) enable automated EB generation in a 96-well format and EB transfer for downstream applications (e.g., into 96-well imaging microplates); (5) introduce a superior routine for flow-cytometric quantification of marker expression; and (6) utilize physiologically more relevant human induced pluripotent stem cells and isogenic primary human fibroblasts for the assay procedure. The proposed workflow was validated using a panel of well-characterized non-embryotoxic, weakly embryotoxic, and strongly embryotoxic compounds.

## Results

### Duration of the assay procedure and compound characteristics

**Fig. 1:**
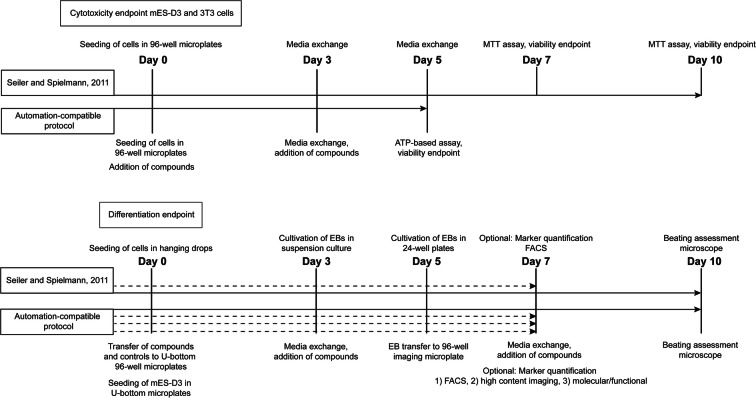
Overview of the EST assay procedure. Overview of steps involved for the completion of the EST (according to (Seiler and Spielmann 2011)) and for the automation-compatible protocol suggested here and the respected time needed

**Fig. 2:**
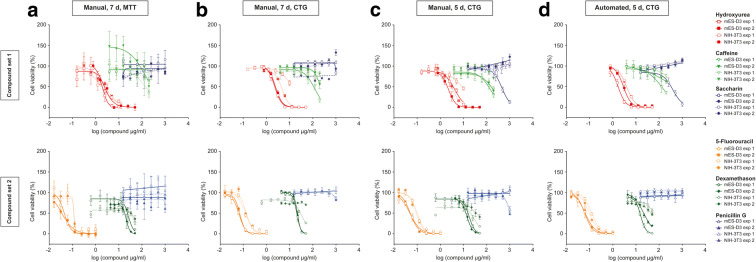
Step-wise evolution of the mESC-D3 and NIH/3 T3 cytotoxicity assay towards assay robustness and high throughput compatibility. Cytotoxicity assays were carried out with two independent compound sets on mESC-D3 and NIH/3 T3 cells in parallel. Set 1 consists of hydroxyurea, caffeine and saccharin (upper row), set 2 of 5-fluorouracil, dexamethason and penicillin G (lower row). (**a**) Conventional 7-day MTT assay and manual handling according to (Seiler and Spielmann 2011). (**b**) As in **a**, but using CellTiter-Glo® viability assay. (**c**) As in **b**, but assay time reduced to 5 days. (**d**) As in **c**, but using an automated procedure. The plate layout as well as the number of wells per compound concentration was set up according to the standard EST protocol (Seiler and Spielmann 2011). All points in the graph represent the mean of 6 individual data points and two individual experiments are plotted, including the standard error. The change of the viability detection system, the assay time, and the introduction of an automated assay routine increased assay performance and reproducibility of data. Corresponding IC_50_ values are summarized in Table S1

To shorten the experimental time without loss of assay performance, we first reduced the duration of drug treatment. Previously developed EST methods required a chemical exposure time of 7–10 days for assessing the viability endpoint (Seiler and Spielmann 2011). Our optimization resulted in the reduction of assay time from 7 to 5 days (Fig. 1). Cell viability was measured after exposure to a defined set of chemicals for both 7 and 5 days and there was no notable difference in IC_50_ measurements between these experimental procedures and the IC_50_ values were in line with EST data obtained in certified pre-validation studies (Scholz et al. 1999) (Fig. 2 and Table S1). All compounds analyzed (non-embryotoxic: saccharin, penicillin G; weakly embryotoxic: caffeine and dexamethasone; strongly embryotoxic: 5-fluorouracil and hydroxyurea) were classified correctly for all experiments conducted (Table S1). Since penicillin G does not lead to impaired fertility or harm to the developing fetus (in mice, rats, rabbits), the viability of the cells should be unaffected. Decades of human experience with the penicillins during pregnancy have not shown any positive evidence of adverse effects on the fetus.

### Homogeneous assay formats for viability assessment based on ATP

In order to assess the cytotoxicity endpoint of the compound-treated mESC-D3 and NIH/3T3 cells, the ATP-based CellTiter-Glo® luminescence cell viability assay was evaluated for a more robust assay alternative compared to the absorbance-based MTT assay. Furthermore, results of the CellTiter-Glo® assay were compared after 5 and 7 days of compound treatment, resulting in similar IC_50_ values between shorter or longer exposure times (Fig. 2 and Table S1). We found that comparable IC_50_ values for compound sets 1 (hydroxyurea, caffeine, saccharin) and 2 (5-fluorouracil, dexamethasone, penicillin G) were determined with both readouts, nevertheless, much higher variability in the data was observed in the MTT assay (Fig. 2, Table S1), which is likely due to the multi-step, time consuming and intrinsic toxic assay format. Furthermore, based on the six compounds analyzed, no differences in the assay performed were observed between longer or shorter assay protocols and a correct compound classification according to consensus calculations (Seiler and Spielmann 2011) could be achieved with all assay procedures (Fig. 2, Table S1). Therefore, the 5-day incubation period in combination with the adaptation to non-toxic homogeneous “mix-and-read” procedures based on ATP and not NADH as a substrate was considered sufficient for implementation of an automated workflow.

### Cytotoxic endpoint based on human-induced pluripotent stem cells and human fibroblasts

The cytotoxicity assessment with a reduced assay time of 5 days combined with either NADH- or ATP-based detection was also carried out with human induced pluripotent stem cells (hiPSCs). We utilized the cell line ZIPi013-E which was shown to give rise to a variety of functional cell types of ectodermal, mesodermal, or endodermal origin by spontaneous differentiation or directed differentiation approaches (Tandon et al. 2018). These hiPSCs were treated with standard compounds from set 1 (hydroxyurea, caffeine) and set 2 (5-fluorouracil, penicillin G), but also the corresponding genetically identical fibroblasts from which the hiPS cells were derived from, namely human dermal fibroblasts of fetal origin (HDFf). The cytotoxicity data obtained from these experiments resulted in comparable IC_50_ values for ZIPi013-E compared with mESC-D3 and HDFf compared with NIH/3T3 cells, although the hiPSCs showed an overall higher sensitivity to the toxicants. The hiPSCs were more sensitive to caffeine (ZIPi013-E, IC_50_ 43.63 μg/ml; mESC-D3, IC_50_ 138 μg/ml), to hydroxyurea (ZIPi013-E, IC_50_ 0.8652 μg/ml; mESC-D3, IC_50_ 2.28 μg/ml) and to 5-fluorouracil (ZIPi013-E, IC_50_ 0.0166 μg/ml; mESC-D3, IC_50_ 0.0627 μg/ml), hinting to a generally higher sensitivity of the human cells (Fig. S1 and Table S2). This increase in sensitivity did not manifest in the corresponding HDFf cells compared with the NIH/3T3 fibroblasts (caffeine HDFf, IC_50_ 411.8 μg/ml; NIH/3T3, IC_50_ > 200 μg/ml; hydroxyurea HDFf, IC_50_ 30.15 μg/ml; NIH/3T3, IC_50_ 4.01 μg/ml; 5-fluorouracil HDFf, IC_50_ 0.1864 μg/ml; NIH/3T3, IC_50_ 0.11 μg/ml). Furthermore, differences in the cytotoxicity effects between the hiPSCs and HDFfs were more pronounced than in the mouse cells. Similar to the cytotoxicity-based assay the overall variance in the data is reduced when viability data was obtained by an ATP-based read-out. In summary, these results pave the way towards an EST solely based on human cells.

### Automation of the cytotoxicity assay for mESC-D3 and NIH/3T3 cells

**Fig. 3:**
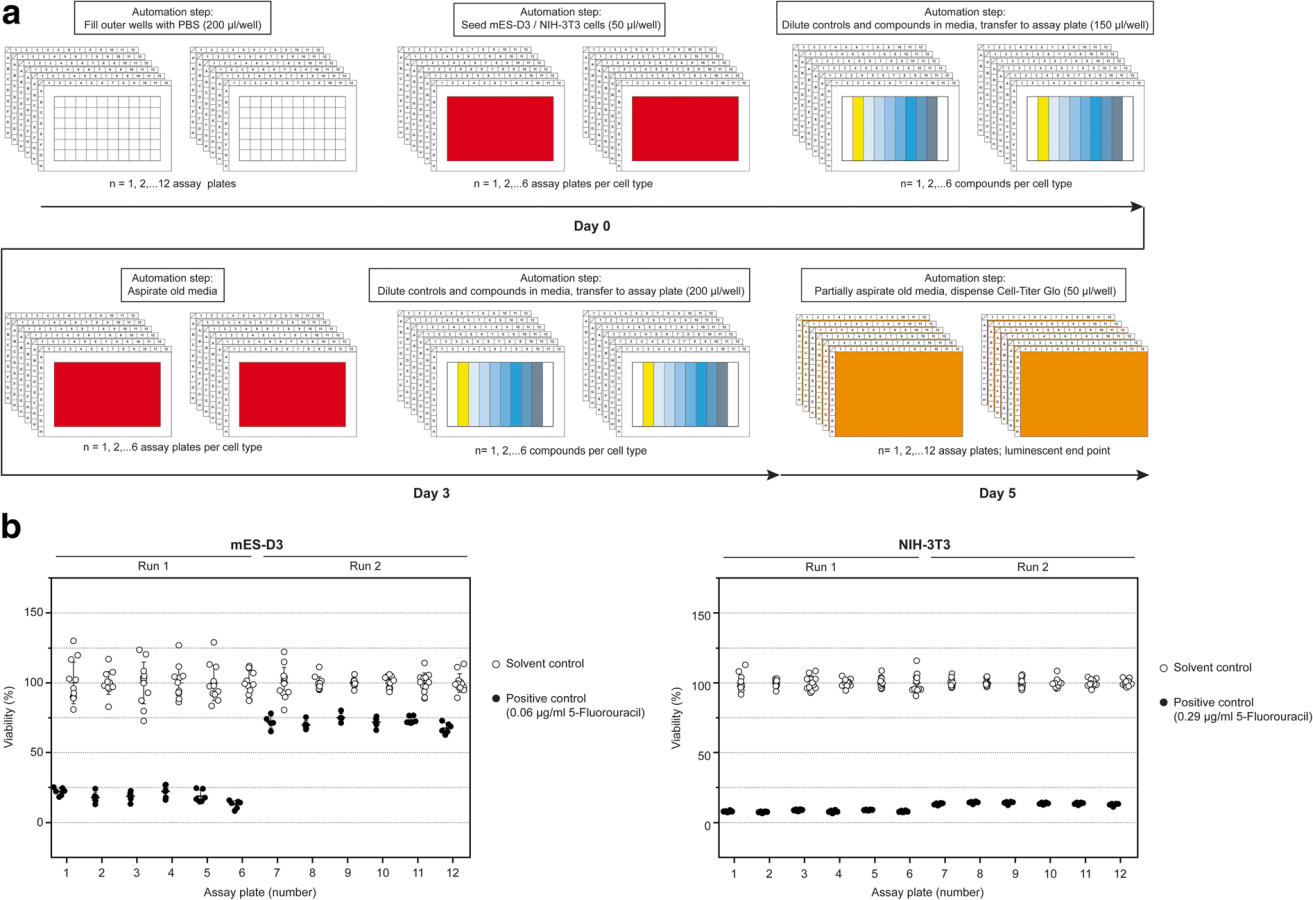
Lab automation for EST-cytotoxicity endpoint. (**a**) Description of automation scripts for sequential procedures for toxicity assessment and associated timeline. (**b**) Plate statistics of solvent and positive control for toxicity assessment. Intra-batch variability of the treatment effect is very low. Inter-batch effects of the positive control on mESC-D3, however, can be observed. Corresponding IC_50_ values are summarized in Table 1

The cytotoxicity assay was adapted to a lab automation platform (Tecan Fluent®). Apart from the preparation of the cell suspension and initial compound dilution, all steps of the assay were adapted to automation routines. Four different building blocks were implemented for automation: Cell seeding, compound dilution and transfer, media exchange, and the CellTiter-Glo® viability assay. To maximize compound throughput, the methods were set up for testing of up to 6 compounds in mESC-D3 and NIH/3T3 cells in parallel, resulting in total in up to 12 plates being processed by the automation system (Fig. 3A). One advantage of this approach is that the same compound dilutions can be applied to both cell lines in parallel, leading to a reduction in intra-assay variability (Fig. 3B). Comparable results were obtained in two independent automation runs, which were also in line with the results obtained in the 5-day manual cytotoxicity assay (Fig. 3B, Table 1). Of note, we observed some inter-assay variability (but little intra-assay variability) of the positive control 5-fluorouracil, which is contained on each microplate analyzed (Fig. 3B, left).

**Table 1:** Results of the automated mouse EST assay

	Cytotoxicity assay		Differentiation assay in round bottom ULA microplate	Compound classification (Seiler and Spielmann 2011)
	CTG, 5 d			
	IC_50_ (μg/ml) mESC-D3	IC_50_ (μg/ml) NIH/3 T3	ID_50_ (μg/ml) mESC-D3	
Saccharin	393 (393; > 1000)	> 1000 (> 1000; > 1000)	> 1000 (> 1000; > 1000)	No embryotoxicity
Penicillin G	> 1000 (> 1000; > 1000)	> 1000 (> 1000; > 1000)	Not determined	Not classified
Caffeine	138 (138; > 200)	> 200 (> 200; > 200)	> 200 (> 200; > 200)	Weak embryotoxicity
Dexamethason	18.7 (15.8; 21.6)	> 50 (> 50; > 50)	Not determined	Not classified
Hydroxyurea	2.28 (1.69; 2.87)	4.01 (3.97; 4.06)	2.69 (2.86; 2.52)	Strong embryotoxicity
5-Fluorouracil	0.0627 (0.0611; 0.0643)	0.11 (0.123; 0.0974)	Not determined	Not classified

### Embryoid body formation in 96-well round-bottom ultra-low attachment plates

**Fig. 4:**
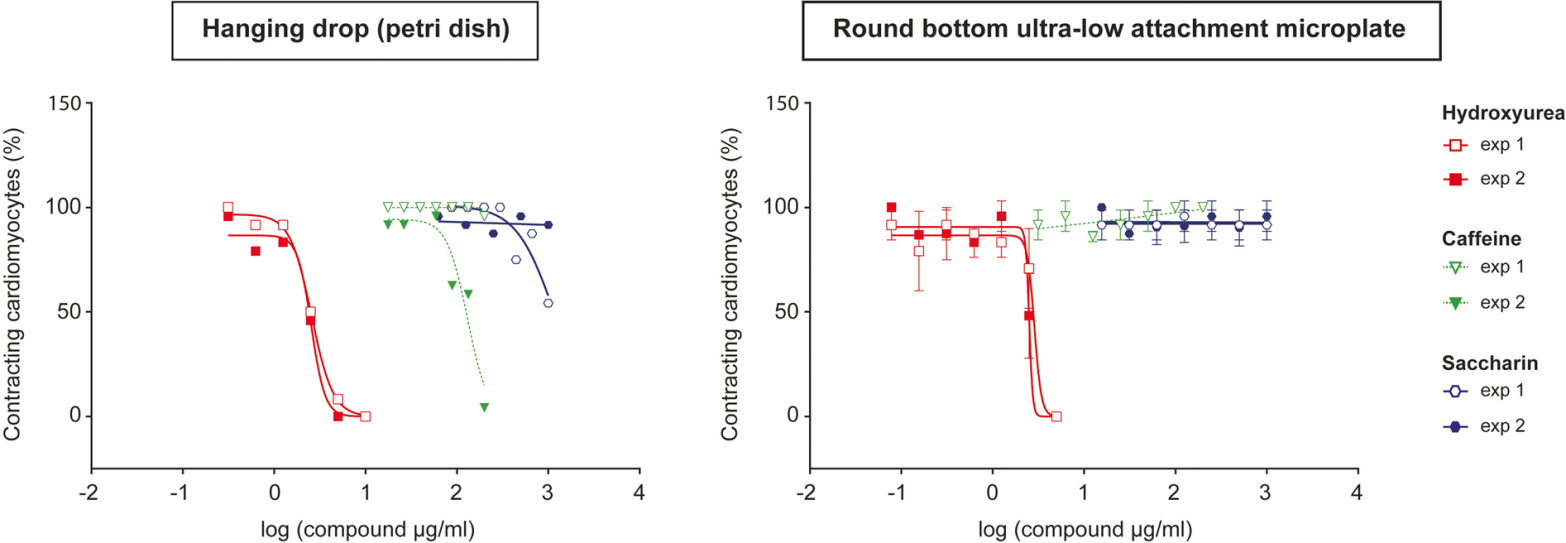
Cardiomyocyte differentiation of mESC-D3 cells in the presence of a compound set 1 (hydroxyurea, caffeine, saccharin). Embryoid bodies were formed in Petri dishes in hanging drops (**a**) or in 96-well round-bottom ultra-low attachment microplates (**b**) and differentiated over 10 days. Cardiomyocyte contraction was detected under the microscope as an endpoint readout. For each data point, 24 sample-containing wells were analyzed. These wells were either located on the same 24-well plates (**a**) or distributed across three 96-well plates (**b**). In the latter case, the mean and standard error is displayed. Two independent experimental repeats are shown for both differentiation procedures

To enable automated liquid handling, a protocol for EB formation in 96-well round-bottom ultra-low attachment plates was set up as an alternative to the classical EB formation protocol in hanging drops in a Petri dish. We compared the assay performances with regard to the functional endpoint of wells containing beating mESC-derived cardiomyocytes after 10 days of exposure to reference compounds. Petri dish and 96-well round-bottom ultra-low attachment-derived EBs generated showed comparable ID_50_ values when treated with compound set 1 (saccharin, caffeine, and hydroxyurea), albeit data from the latter showed less intra-assay variability (Fig. 4, Table 1, Table S1), presumably due to the formation of more standardized EBs. Therefore, EB formation via 96-well round-bottom ultra-low attachment plates was considered sufficient for the generation of cells that can be interrogated for the functional endpoint and that justify the implementation of an automated workflow.

### Automation of embryoid body formation in U-bottom plates and downstream analysis options

**Fig. 5:**
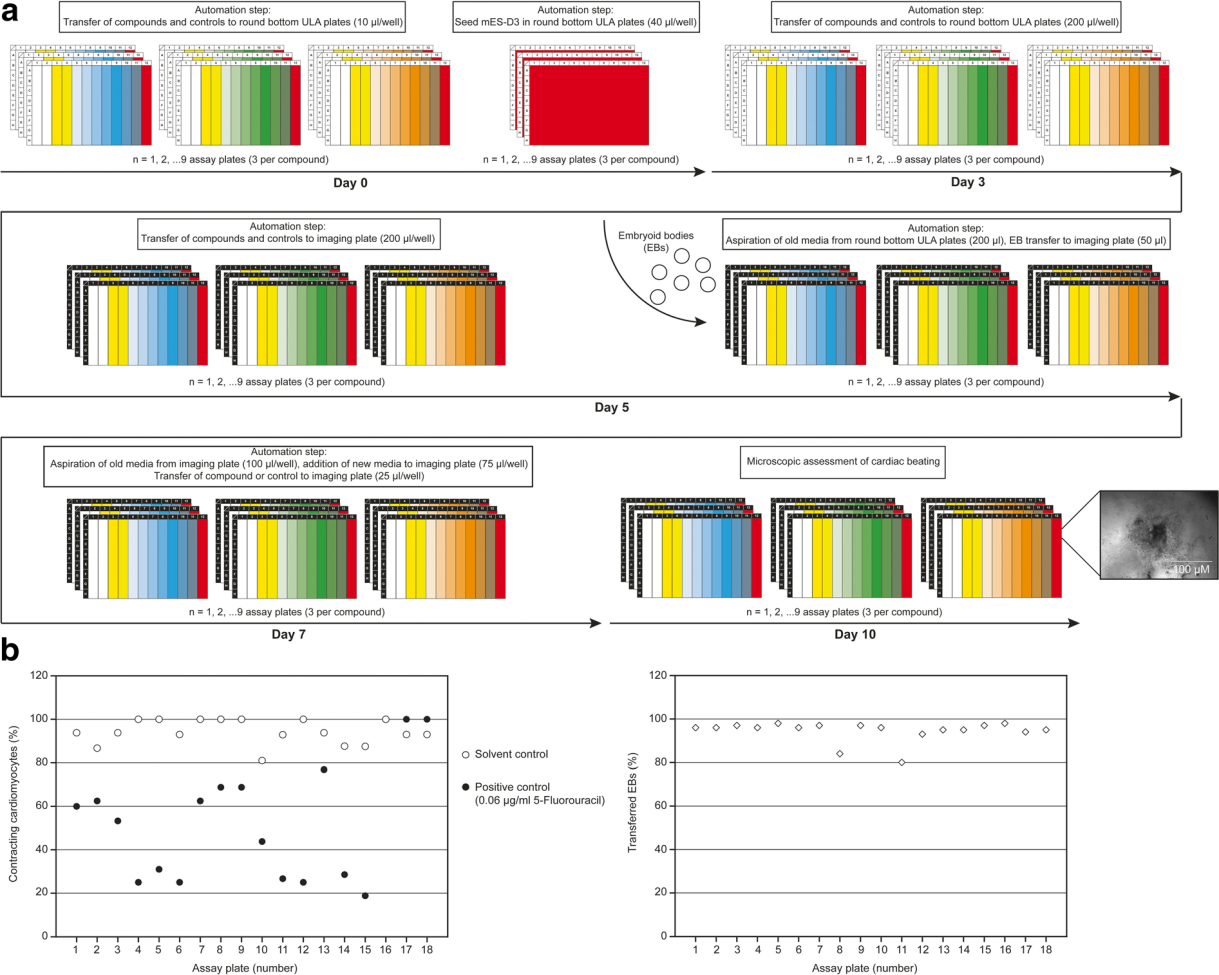
Lab automation for EST–differentiation endpoint. (**a**) Description of automation scripts for sequential procedures for differentiation assessment and associated timeline. (**b**) Plate statistics of solvent and positive control for differentiation assessment and embryoid body transfer rate. Due to cut-off criteria, assay plates 16–18 were invalidated and therefore excluded from further analysis. EB transfer efficiency was reproducibly high (> 80%)

The procedure for the generation, differentiation and analysis of EBs was adapted to a lab automation platform (Tecan Fluent®). Four different building blocks were implemented for automation: Cell seeding, compound dilution and transfer, media exchange, and EB transfer to 96-well imaging microplates. To maximize compound throughput, the methods were set up for testing of up to 3 compounds in mESC-D3 in parallel for three optional endpoints: (1) microscopic assessment of mESC-derived beating cardiomyocytes, (2) quantitative analysis of marker expression, (3) and optional third parameter customizable by the user. This requirement resulted in a total of up to 9 plates being processed in parallel by the automation system (Fig. 5A). One advantage of this approach is that the same compound dilutions can be applied to all assay plates in parallel, leading to a reduction in intra-assay variability, although some inter-assay variability with regard to the performance of the positive control (5-fluorouracil) can be observed (Fig. 5B). Comparable results were obtained in independent runs analyzing 18 microplates. At this stage, assay plates could easily be excluded from further experiments if the positive control was ineffective (here plates 17 and 18, Fig. 5B). Of note, the rate of successfully transferred EBs in this automated procedure was high across 18 assay plates, with only two plates with an EB transfer rate below 90% (Fig. 5B, right).

### Molecular differentiation endpoint-optimization of marker expression analysis by flow cytometry

**Fig. 6:**
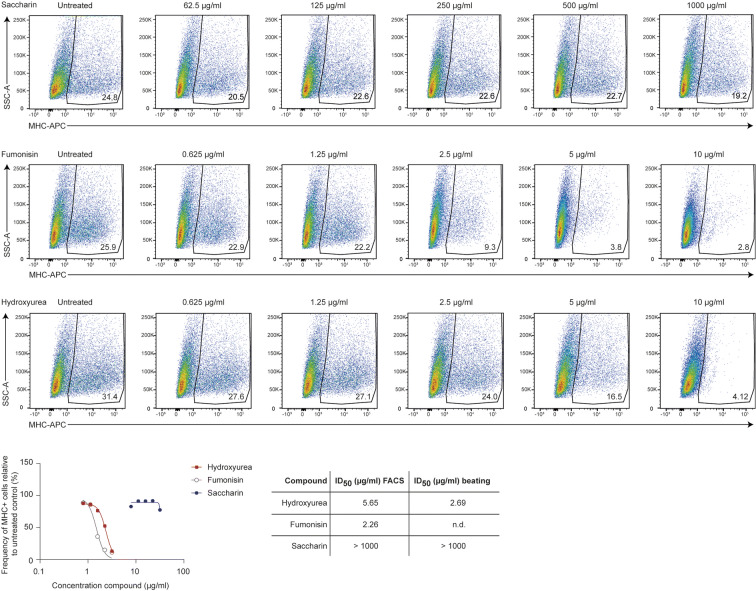
Flow-EST. Assessment of the differentiation endpoint of the EST assay for saccharin (non-embryotoxic), fumonisin (strongly embryotoxic), and hydroxyurea (strongly embryotoxic) by flow cytometry using a directly labeled MHC-specific antibody. While Saccharin does not impact the differentiation process (top panel), the impact of both fumonisin and hydroxyurea is pronounced. ID_50_ results for hydroxyurea and saccharin are in line with data obtained from the conventional beating analysis

As an alternative to the morphological analysis at day 10, differentiation can be analyzed at day 7 of the assay by flow cytometry using the molecular flow EST (Buesen et al. 2009). Previously, staining of cardiomyocytes was performed using an unconjugated primary monoclonal antibody directed against the sarcomeric MHC (clone MF20), followed by binding of a secondary biotin-conjugated goat anti-mouse IgG antibody which is then detected with the fluorochrome PE-SA (phycoerythrin-conjugated streptavidin) (Seiler and Spielmann 2011). We here describe an improvement of this workflow using (1) more gentle dissociation reagents to prevent cell death during the preparation of the samples, (2) incorporation of live/dead staining to gate out dead cells, and (3) direct labeling of the cardiac-specific marker protein sarcomeric MHC with fluorophore-coupled antibodies. We started off the optimization by recapitulating the dissociation and staining procedure according to established protocols (Seiler and Spielmann 2011), resulting in low amounts of living, MHC-positive cells (Fig. S2, upper panel). Cell survival could be increased significantly by the introduction of more gentle dissociation reagents. Furthermore, by use of live/dead staining reagents and accordingly exclusion of dead cells from the analysis, we could increase the relative percentage of MHC-positive cells among living cells as readout parameter to ~ 50% (Fig. S2, lower panel). Of note, these experiments were performed in parallel with the same batch of spontaneously differentiated mESCs. Using this new isolation, staining, and gating strategy (Fig. S3), we exposed the cells to the well-characterized strongly embryotoxic compounds hydroxyurea or fumonisin or to the non-embryotoxic control saccharin (Fig. 6). While saccharin exposure reduced the amount of MHC-positive cells only slightly (25% untreated vs. 19% treated with 1000 μg/ml, ID_50_ > 1000 μg/ml), exposure to hydroxyurea (31% untreated vs. 4% treated with 10 μg/ml, ID_50_ 5.65 μg/ml) had a profound and dose-dependent effect on the frequency of MHC-positive cells (Fig. 6). Both ID_50_ values are in line with the compound-mediated effects on cardiac beating in the functional assay (hydroxyurea 2.69 μg/ml, saccharin > 1000 μg/ml), therefore molecular read-outs at day 7 could be used as a surrogate for the functional beating assay on day 10. Of note, the mycotoxin fumonisine B1, a modulator of sphingosine metabolism, also led to a profound and dose-dependent reduction in MHC-positive cells (26% untreated vs. 3% treated with 10 μg/ml, ID_50_ 2.26 μg/ml) (Fig. 6). We are therefore confident that the modified flow cytometry procedure is able to reliably assess the impact of embryotoxic compounds on molecular marker expression in embryoid body derived cells.

### Molecular differentiation endpoint-marker expression analysis by high content imaging

Differentiation capacity of the cells can be analyzed at day 7 of the workflow, but flow cytometric analysis of marker expression is only one potential end-point assay. Alternatively, high content imaging using confocal microscopy can be performed. To enable high content imaging studies, the EBs from the 96-well round bottom ultra-low attachment microplates were automatically transferred to the pre-coated 96-well imaging plates on day 5 and the cells were cultivated for another 2 days, resulting in outgrowth from the EB. Fixation of the EBs and successive staining with nuclear dyes (Hoechst 33342) and cardiomyocyte-specific markers (e.g., alpha-Actinin) enables whole well imaging or imaging of a distinct number of image fields with a high content imaging device. Wells without nuclear staining can be excluded from further analysis (Fig. S4B, left panel, black dots). Toxic substances in high concentrations, however, also produce a similarly low nuclear staining signal than a well with no EB transferred (Fig. S4B, right panel). Therefore, high content imaging approaches might be in particular suited for the testing of weakly embryotoxic compounds or low concentrations of toxic compounds. In higher magnification, EB outgrowths are alpha-Actinin positive and show sarcomeric structures (Fig. S4A), which can be quantified. Alpha-Actinin positivity, however, does not correlate with the ability of an EB to show beating (data not shown).

## Discussion

Cell-based screenings for toxicity assessment are a necessary tool in all stages of pre-clinical development and/or market approval of new drugs and chemicals today (Shinde et al. 2016). The major change over the last decade is a shift towards more physiologically relevant yet complex and sensitive cell models, based on embryonic stem cells, and more recently, based on human-induced pluripotent cells (hiPSCs). Screening technologies using pluripotent cells have the potential to revolutionize drug discovery by allowing in vitro direct testing of pathways of embryogenesis even in primary screens, safety pharmacology, metabolic profiling, and toxicity evaluation (Engle and Puppala 2013; Hosoya and Czysz 2016; Pouton and Haynes 2007). In addition, toxicological safety assessments for chemicals and drugs are legally required to investigate possible chemical-induced developmental or reproductive toxicity. Alone within the REACH program (Registration, Evaluation, Authorisation & Restriction of CHemical substances) > 100,000 chemicals have to be evaluated according to their toxicological profile. The most demanding studies are in the area of reproductive toxicity testing with about 90% of all animal use and 70% of the required costs for registration. The overall result suggests a demand of 54 million vertebrate animals and testing costs of 9.5 billion Euro apart from the ethical issue (Rovida and Hartung 2009). This clearly challenges the feasibility of the REACH program without a major investment into high-throughput methodologies to significantly reduce the use of animals for toxicology testing. Consequently, the development of rapid and predictive cell-based in vitro screening methods with the potential to replace the current animal models is a focus of research. The screening methods required need to be selected and evolved towards automation-compatibility, miniaturization, and reproducibility to achieve high standardization along with low material consumption. Application of these standardized methods could lead to (a) a reduction in the number of experimental animals, (b) a shortening of the turnaround time to assess compound characteristics, and (c) a resulting increase in cost-effectiveness of the overall process. Ideally, these in vitro tests should increase the predictive power by the introduction of more reliable and physiologically more relevant assays based on human cells.

To date, the mouse EST remains the most thoroughly validated ESC-based assay for developmental toxicant testing. It has undergone thorough validation studies coordinated by the European Center for the Validation of Alternative Methods (ECVAM) (Genschow et al. 2004; Genschow et al. 2002). However, it is also important to note that assay validation does not equate to regulatory acceptance, and to date, no assay based on pluripotent stem cells is used to make regulatory decisions. Since its validation, the mouse EST has been used extensively in academia and pharma to test the embryotoxic potential of a wide array of compounds, including pharmaceuticals (Eckardt and Stahlmann 2010; Paquette et al. 2008), industrial chemicals (de Jong et al. 2009), cosmetics (Chen et al. 2010), nanoparticles (Di Guglielmo et al. 2010; Park et al. 2009), metals (Stummann et al. 2007, 2008), and various environmental contaminants (Kamelia et al. 2017; Kong et al. 2013; Zhou et al. 2017).

To predict the developmental toxicity of a given test substance in the EST, three endpoints are used: (a) The cytotoxic effect on stem cells, (b) the cytotoxic effects on NIH/3T3 fibroblasts, and (c) inhibition of mESC differentiation into beating cardiomyocytes (Seiler and Spielmann 2011). Further distribution and rigorous application of the validated and sophisticated EST test for toxicological safety assessment was hindered by a number of factors: (1) Manual processing of a complex protocol which has to handle both the 2D cultivation regime and the “hanging drop” cultivation method developed for neural tissue culture in the early twentieth century. It is obvious that this test scenario is not designed for automated, operator-free processing and is essentially incompatible with the requirements of high-throughput screening in its current form (Peters et al. 2008). We have therefore optimized the assay towards the generation of standardized EBs in 96-well microplate formats accompanied by automated liquid handling for media and compound exchange and EB transfer for downstream analysis. The long-term processing times of 10 days demanding extraordinary robustness combined with complex readout decisions depending on high-content information. We addressed this by reducing the overall assay time where feasible combined with a reduction in the number of times media/compound was changed. The period of drug exposure has now been changed to 5 days in total, which reduces errors and costs associated with media changes paired with an increase in accuracy of the assay. Of note, it could be desirable to assess toxicity parameters at different time points after compound exposure. The automated cytotoxicity assay suggested here can easily be customized by addition of implemented sub-scripts to (a) replenish compounds or (b) perform viability measurements based on ATP at any point in time, as summarized graphically in Fig. 3. We have also implemented scripts utilizing assays to monitor cell viability continually in the same sample well up to 72 h to assess time and dose dependent parameters in one run (e.g., RealTime-Glo™, Promega), indicating the flexibility of the approach.

As a read-out for the differentiation endpoint, high content imaging based on confocal microscopy can be performed and we have conducted this based on the expression on the cardiomyocyte-specific marker alpha-Actinin. Apart from this, other markers have been suggested as end-point parameters, for example the reduction in nuclear translocation of the transcription factor SOX17 during direct differentiation into mesendodermal cells, since decreased nuclear SOX17 was strongly correlated with in vivo teratogenicity (Kameoka et al. 2014). Kang et al. have suggested to directly assess the EB area, since a decline in EB area after compound exposure correlated with a decrease of beating frequency during differentiation of PSCs (Kang et al. 2017). All in all, a molecular marker which shows strong correlation with the EB’s beating capacity would be highly desirable to further shorten the assay procedure to interrogate the differentiation endpoint.

In order to evaluate our procedure, we tested and compared a panel of six well-characterized compounds in the assays developed. From the group of well-characterized non-embryotoxic compounds saccharin and penicillin G were chosen, from the group of weakly embryotoxic compounds caffeine and dexamethasone were selected and from the group of strongly embryotoxic compounds 5-fluoruracil and hydroxyurea were evaluated. These chemicals were considered as representative for each class in previous prevalidation studies (Scholz et al. 1999).

In summary, we have adapted the ECVAM validated EST to shorter assay time periods, homogenous viability assay formats, and a superior marker quantification routine. Most importantly, we have automated essential parts of the workflow (cell seeding, media exchange, compound dilution and distribution, viability assessment, EB transfer) and also enable alternative molecular/functional endpoints, e.g., flow cytometry and high content imaging. Furthermore, we provide initial evidence that the procedure could also be adapted to human induced pluripotent cells and their isogenic reference cell lines, providing a physiologically more relevant assay technology for future use, potentially also for personalized toxicity studies.

## Experimental procedures

### Cell maintenance

For culturing mESC-D3 (ATCC® CRL-11632™) culture vessels were coated with gelatin (0.1%). mESC-D3 growth media (DMEM high glucose, 20% fetal bovine serum (Capricorn Scientific, Cat. No. FBS-ES-22A), 2 mM L-glutamine, 500 Units/ml penicillin, 0.1 mg/ml streptomycin, 1% non-essential amino acids (Life Technologies, Cat. No. 11 140-035), 0.1 mM 2-mercaptoethanol (Cat. No. 21985–023)) and NIH/3T3 (ATCC® CRL-1658) growth media (DMEM high glucose, 10% fetal bovine serum, 4 mM L-glutamine, 500 Units/ml penicillin, 0.1 mg/ml streptomycin) were used no longer than 1 week after preparation. For cell passaging the media was removed from the culture vessel and cells were washed once with pre-warmed Dulbecco’s PBS (Capricorn Scientific, Cat. No. PBS-1A). Afterward, 5 ml Trypsin were dispensed into the culture vessel (75 cm^2^). As soon as the cell layer was dispersed, a 10-ml culture medium was added to stop the reaction and the cell suspension was transferred into a 50-ml tube. The tube was centrifuged 5 min at 300×*g*. The supernatant was discarded and the cell pellet was suspended in fresh pre-warmed culture media. A fraction of the cell suspension was transferred into a new cell culture vessel. Leukemia Inhibitory Factor (Millipore, ESG1107, stock 10^7^ Units, 1:1000 dilution) was added directly to the mESC-D3 culture vessel in order to maintain the cells in an undifferentiated state. HDFf cells (ScienCell, Cat. No. #2300) were cultivated in Fibroblast Medium (ScienCell, Cat. No. #2301) and were used for derivation of human iPSCs (ZIPi013-B and ZIPi013-E) (Tandon et al. 2018). ZIPi013-E were used for this study and maintained under feeder-free conditions on Matrigel (Corning)-coated plates in mTeSR1 medium (Stem Cell Technologies, Cat. No. #85850). The medium was exchanged on a daily basis and cells were passaged at 80% confluency using gentle cell dissociation reagent (Stem Cell Technologies, Cat. No. #07174). All cells were incubated at 37 °C, 5% CO_2_, and 95% humidified atmosphere.

### MTT cell viability assay

The MTT cell viability assay was performed as described (Seiler and Spielmann 2011) with modifications. In brief, 96-well cell culture plates were coated with gelatin (0.1%) before plating mESC-D3. All peripheral wells of the assay plate were filled with 200 μl growth media (“blank”). Afterward, NIH/3T3 and mESC-D3 were plated in growth media (1 × 10^4^ cells/ml, 50 μl/well). A 7-point dilution series in growth media was prepared for each test compound (hydroxyurea (Sigma, Cat. No. H8627-1G), 5-fluorouracil (Sigma, Cat. No. F6627-5G), fumonisin (VWR, Cat. No. SAFSF1147-1MG), caffeine (Sigma, Cat. No. C0750-5G), dexamethasone (Sigma, Cat. No. D4902-25MG), penicillin-G (Sigma, Cat. No. PENNA-100MU), saccharin (Sigma, Cat. No. S1002-500G)), and was transferred to the assay plate 2 h after cell seeding (150 μl/well). Vehicle (PBS) and positive control (5-fluorouracil) wells were also included on all assay plates. Three and 5 days after seeding the cell supernatant was replaced by freshly prepared compound solution (200 μl/well). Seven days after seeding the cell viability was detected. Therefore, the cell supernatant was aspirated from all wells, MTT solution (5 mg/ml) was added (20 μl/well) and the assay plate was incubated for 2 h at 37 °C and 5% CO_2_ in a humidified atmosphere. Afterward, all liquid was removed from the wells and MTT desorb solution (0.7% SDS in 2-propanol (Carl Roth, Cat. No. 9866.5), pre-warmed to 37 °C) was dispensed (130 μl/well). The assay plate was placed on a shaker for 15 min. Remaining precipitates were suspended by pipetting up and down with a multichannel pipette before measuring the absorption at 570 nm on a microtiter plate reader using 630 nm as a reference wavelength.

### CellTiter-Glo® luminescent cell viability assay

96-well cell culture plates were coated with gelatin (0.1%) before plating mESC-D3. All peripheral wells of the assay plate were filled with 200 μl growth media. In order to assess compound toxicity on mESC-D3 and NIH/3T3 cells, cell suspensions of NIH/3T3, and mESC-D3 were prepared in growth media (1 × 10^4^ cells/ml) and were transferred to all remaining wells (50 μl/well). A 7-point dilution series in growth media was prepared for each test compound and was transferred to the assay plate 2 h after cell seeding (150 μl/well). Afterward, the cells were incubated in a humidified atmosphere at 37 °C and 5% CO_2_. Three and 5 days after seeding the cell supernatant was replaced by freshly prepared compound solution (200 μl/well). Seven days after seeding the assay plate was taken out of the incubator and located at room temperature for 10 min, before cell supernatant was aspirated (80 μl/well) and CellTiter-Glo® reagent was dispensed (50 μl/well). The assay plate was placed on a shaker for 1 min and after another 10 min incubation at room temperature in the dark the luminescence signal was detected on a plate reader.

### Conventional mouse ES-D3 differentiation assay in hanging drop format

The mESC-D3 differentiation assay was performed according to (Seiler and Spielmann 2011). A 7-point dilution series was prepared in growth media for each test compound. A suspension of mESC-D3 (3.75 × 10^4^ cells/ml) was mixed with the compound solution. Of the cell suspension with test compound (= 750 cells/drop), 20 μl drops were placed on the underside of a 100-mm Ø tissue culture Petri dish lid, at minimum of 40 drops per lid. Separate Petri dishes were used for each concentration of test compound, untreated control, and solvent control. The Petri dish was filled with 5 ml PBS and the lid was carefully turned and put on top. After 3 days of incubation at 37 °C and 5% CO_2_ in a humidified atmosphere, the EBs were rinsed from the lid with 5 ml of the freshly prepared compound solution into a 60-mm Ø bacterial Petri dish. The EB suspension was incubated for 2 days. On day five of the assay, a fresh compound dilution series was prepared in media and transferred into 24-well cell culture plates (1 ml/well, Greiner bio-one, Cat. No. 662641). A separate 24-well plate was used for each compound concentration, untreated control, and solvent control (24 wells per condition). One EB was transferred from the Petri dish into each well of the 24-well plate. After further 5 days of incubation (= day 10 of the assay), cardiomyocyte contraction was determined in each well under the light microscope.

### Automated CellTiter-Glo® luminescent cell viability assay

The CellTiter-Glo® luminescent cell viability assay (Promega, Cat. No. G7573) was automated in 96-well format on the lab automation platform Fluent® (Tecan, Männedorf, Switzerland). The procedure allowed the testing of six compounds in mESC-D3 and NIH/3T3 cells in parallel. On the first day, PBS was dispensed into the peripheral wells of the assay plates (200 μl/well) using the Multiple Channel Arm (MCA). Mouse ES-D3 and NIH/3T3 cells were plated (50 μl/well, 1 × 10^4^ cells/ml)) using the Flexible Channel Arm (FCA) and transported into the Cytomat™ 10 (Thermo Fisher Scientific) for incubation using the Robotic Gripper Arm (RGA). A 7-point dilution series of the test compounds and control solutions were prepared in PBS or DMSO (Carl Roth, Cat. No. HN47.1) under the laminar flow, transferred to a 96-well 0.5-ml Masterblock plate (Greiner bio-one, Cat. No. 786261) and placed on the worktable. On the Fluent the compounds were transferred to a 96-well 2-ml Masterblock plate (Greiner bio-one, Cat. No. 780271) and further diluted in growth media using the FCA. Two hours after cell plating compounds and controls were transferred to the assay plate with the FCA (150 μl/well) and transported back in the Cytomat 10. On day three of the assay, the media was exchanged using the MCA: old media was aspirated and dispensed into a 96-deep-well plate (“waste”), freshly prepared compound, and control solution was dispensed into the assay plate. After two more days of incubation, the cell viability was determined using the CellTiter-Glo assay. The assay plates were transported from the Cytomat 10 to the Carousel (Tecan) to adapt to room temperature. After 25 min the cell supernatant was removed partially (80 μl/well) and CellTiter-Glo reagent was dispensed (50 μl/well). The assay plate was placed on a shaker for 1 min and afterward incubated 25 min in the Carousel before the Luminescence signal was detected on the Infinite M1000 Pro plate reader (Tecan).

### Automated mESC-D3 differentiation assay in 96-well round-bottom ultra-low attachment plates

The mESC-D3 differentiation assay was automated in 96-well round-bottom ultra-low attachment plates (Costar, Cat. No. 7007) on the lab automation platform Fluent (Tecan). The method was set up for testing up to three different compounds in an automated procedure, resulting in maximal 9 plates that were being processed. A 7-point dilution series of test compounds and control solutions were prepared in PBS or DMSO under the laminar flow, transferred to a 96-well 0.5 ml Masterblock plate, and placed on the Fluent worktable. There the compounds were further diluted in growth media in a 96-well 2 ml Masterblock plate using the FCA. Of the compound solution, 10 μl/well were dispensed into a 96-well round well ultra-low attachment plate. In the meantime, a suspension of mESC-D3 (3.75 × 10^4^ cells/ ml) was prepared in growth media and was placed in a trough on the worktable. The cell suspension was transferred into the 96-well round-bottom ultra-low attachment plate (40 μl/well) using the FCA. After 2 days of incubation at 37 °C, 5% CO_2_, and 95% humidity fresh compound and control solutions were prepared and added to the 96-well U-bottom plate (200 μl/well). On day five of the assay freshly prepared compound and control solutions were dispensed into a 96-well imaging plate (200 μl/well, Greiner bio-one, Cat. No. 655866). Using the MCA, the supernatant was aspirated from the 96-well round-bottom ultra-low attachment plate, the EB at the bottom of the well was aspirated and transferred into the prefilled imaging plate. On day 7, the cell supernatant was partially replaced with a fresh compound solution. Therefore, the media was aspirated using the MCA and the fresh compound solution was dispensed with the FCA. After further 3 days of incubation (= day 10 of the assay) cardiomyocyte contraction was determined in each well under the light microscope. Afterward, cells were stained and analyzed using the Opera High Content Screening System (PerkinElmer).

### mESC-D3-based flow assay (flow-EST)

Embryoid bodies were formed in hanging drops (Petri dish) as already described. On day 5 of the assay, the EBs were transferred into a larger Petri dish and incubated for two more days at 37 °C, 5% CO_2_, and 95% humidity. Afterward, the EBs were dissociated using the Embryoid Body Dissociation Kit (Miltenyi Biotec, Cat. No. 130-096-348). EBs from two Petri dishes were harvested in one 15 ml tube and washed once with 10 ml PBS. The supernatant was discarded after centrifugation and a pre-warmed enzyme mix was added. The EB/enzyme mix was incubated for 10 min at 37 °C, dissociated for 1 min with a 1000 μl pipette, incubated for 5 min and mechanically dissociated again for 1 min. After the addition of 3 ml PBS, the cell suspension was passed through a 70-μm filter into a new 15-ml tube. The filter was then washed with 3 ml PBS and discarded afterward. The cell suspension was centrifuged for 5 min at 300 rpm. The supernatant was discarded afterward, cells were suspended in PBS and counted. For viability staining, 10^6^ cells were suspended in 100 μl staining reagent (LIVE/DEAD™ Fixable Green Dead Stain Kit, Life Technologies, Cat. No. L34969), incubated for 30 min at 4 °C and washed with 1 ml PBS. For fixation, cells were suspended in 100 μl fixation buffer (BioLegend, Cat. No. 420801) and incubated 20 min at room temperature. For the intracellular staining, cells were washed twice with 1 ml permeabilization buffer (BioLegend, Cat. No. 421002) and suspended in 50 μl permeabilization buffer supplemented with 50 μl of pre-diluted (1:5) antibody (mouse IgG2B anti-human Myosin Heavy Chain APC-conjugated, mouse IgG2B APC-conjugated isotype control, R&D Systems, Cat. No. IC4470A). After 1 h incubation at room temperature cells were washed twice with 1 ml permeabilization buffer and suspended in FACS buffer. Within 2–3 h samples were measured on an LSR II flow cytometer (Beckton Dickinson). Data analysis was done using FlowJo software (Version 10.0.8; Tree Star).

### Data analysis

Toxicity data from the MTT and CellTiter-Glo Assay was normalized against the vehicle control (100% viability) and plotted against the logarithmic compound concentration. Dose-response curves were fitted in Prism (GraphPad) using a four-parameter logistic fit to determine IC_50_ values. For the fitting, bottom values were constrained to 0 for all dose-response assessments. IC_50_ values were rounded down to preferably overestimate rather than underestimate compound activities. The results of the mESC-D3 differentiation assay were expressed as a percentage of wells with contracting cardiomyocytes relative to vehicle control. This data was plotted against the logarithmic compound concentration and dose-response curves were fitted in Prism (GraphPad) using a four-parameter logistic fit to determine the ID_50_. Based on the results from the toxicity and differentiation assays, compounds were classified according to the “validated prediction model of the EST” (Seiler and Spielmann 2011) into three groups: non-embryotoxic, weakly embryotoxic, and strongly embryotoxic.

## Electronic supplementary material

ESM 1 (DOCX 797 kb)

## Data Availability

All authors confirm that all data and materials support their published claims and comply with field standards.
