# Supplementary material for: An automated and high-throughput-screening compatible pluripotent stem cell-based test platform for developmental and reproductive toxicity assessment of small molecule compounds
Source: Cell Biol Toxicol. 2020 Jun 20;37(2):229–43. doi: 10.1007/s10565-020-09538-0 (PMC8012336; doi:10.1007/s10565-020-09538-0)
Supplement: Supplementary file 1 — (DOCX 797 kb) [file 10565_2020_9538_MOESM1_ESM.docx]

**An automated and high-throughput-screening compatible pluripotent stem cell-based test platform for developmental and reproductive toxicity assessment of small molecule compounds**

**Gesa Witt, Oliver Keminer, Jennifer Leu, Rashmi Tandon, Ina Meiser, Anne Willing, Ingo Winschel, Jana-Christin Abt, Björn Brändl, Isabelle Sébastian, Manuel A Friese, Franz-Josef Müller, Julia C Neubauer, Carsten Claussen, Heiko Zimmermann, Philip Gribbon and Ole Pless^§^**

**^§^Corresponding author; ole.pless@ime.fraunhofer.de**

**Supplementary Material**


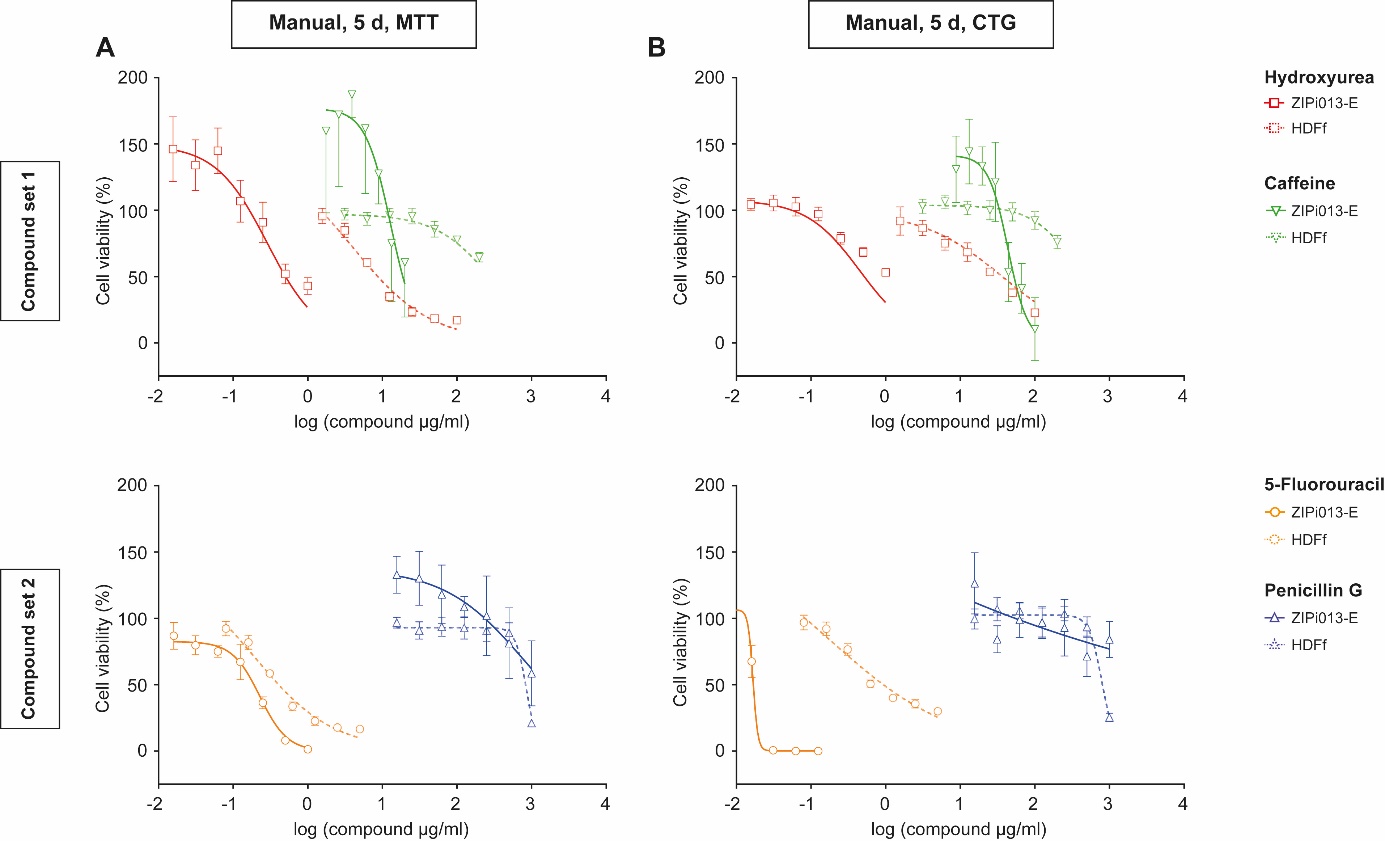


**Figure S1: Step-wise evolution of the cytotoxicity assay towards the use of human cell lines**. Related to Figure 2. Cytotoxicity assays were carried out with two compound sets on hiPSC line ZIPi013-E and HDFf, from which they were derived. Set 1 consists of hydroxyurea and caffeine (upper row), set 2 of 5-fluorouracil and penicillin G (lower row). **A**) 5 day MTT assay and manual handling. **B**) As in A), but using CellTiter-Glo® viability assay. All points in the graph represent the mean of 6 individual data points, including the standard error. ~~The change of the viability detection system significantly increased assay performance and reproducibility of data.~~ Corresponding IC_50_ values are summarized in Table S2.


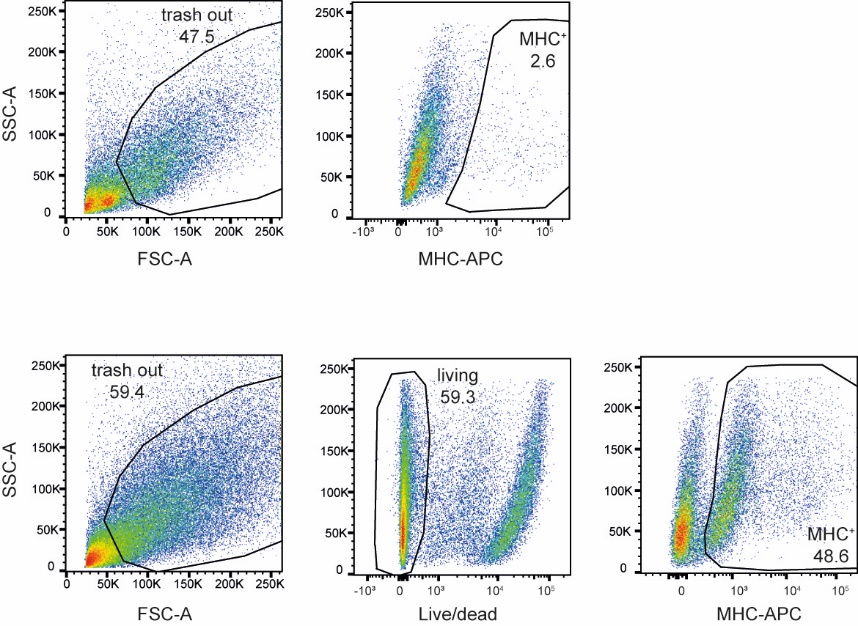


**Figure S2: Evolution of the Flow-EST protocol**. Related to Figure 6. Flow-EST protocol as carried out according to ^1^ (top panel) using rather harsh dissociation conditions results in a low total yield of MHC+ cells in a two-step gating procedure. Introduction of milder cell dissociation reagents and of live/dead staining resulted in a much higher yield and relative percentage of MHC+ cells for assessment of the differentiation endpoint of the EST assay. Of note, both experiments were performed on the same day with the same batch of spontaneously differentiated mESCs.


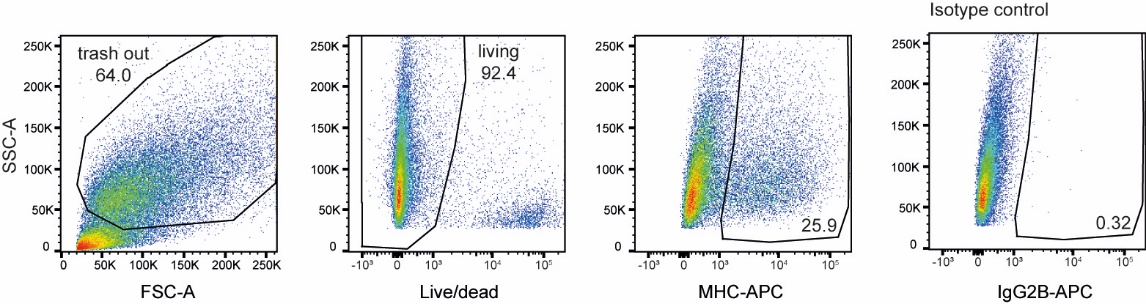


**Figure S3: Gating strategy for Flow-EST**. Related to Figure 6. Flow cytometric identification of MHC+ cells for assessment of the differentiation endpoint of the EST assay. In this experiment, the fraction of MHC+ cells is ~26 %. Almost no positive events were detected when the MHC-specific antibody was replaced by a respective isotype control at equal concentration in the panel (gating on the same population).


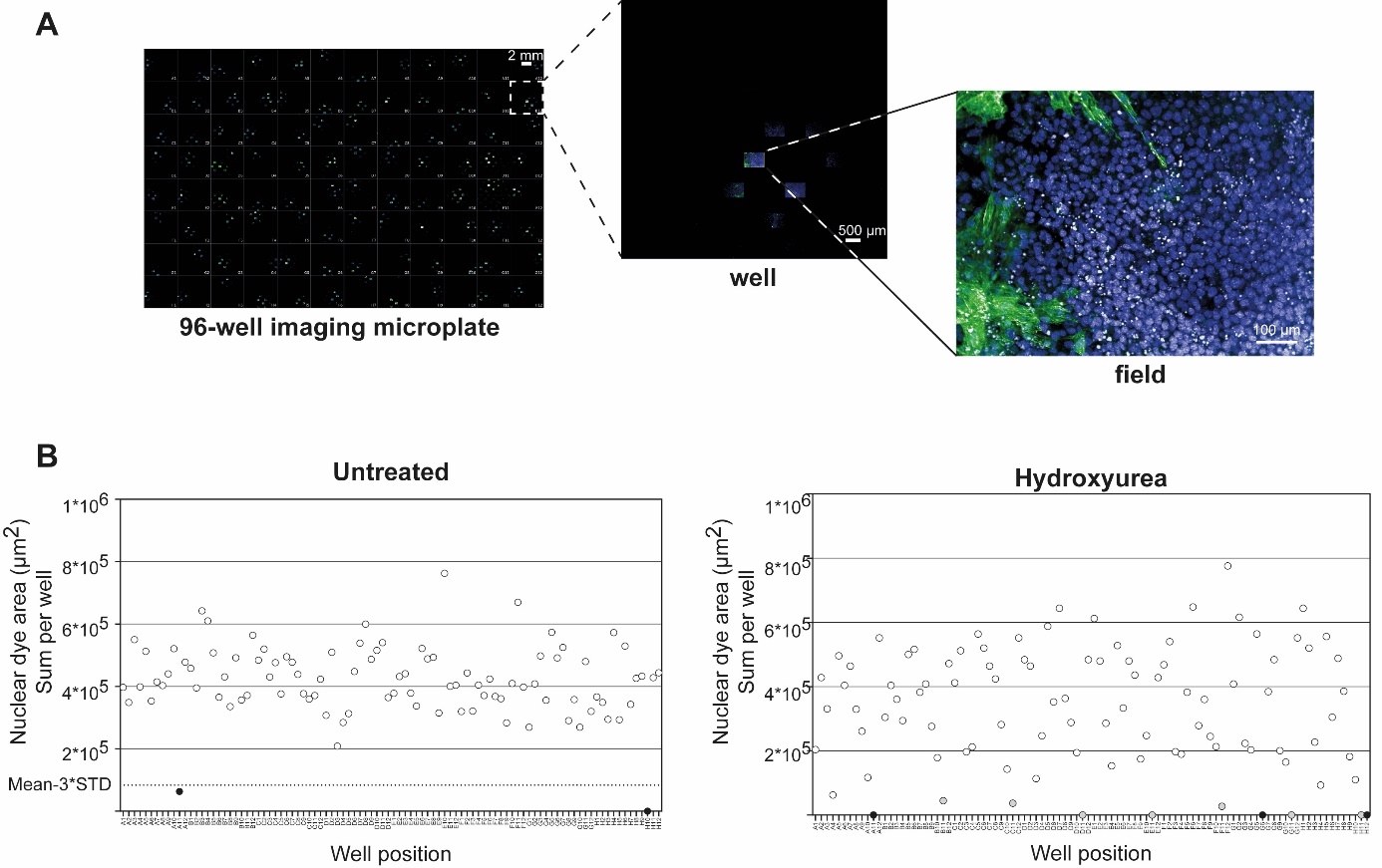


**Figure S4: EB staining in 96-well imaging microplates**. Related to Figure 5. **A**) Schematic images from a suggested workflow for high content imaging applications. After fixing, marker staining (here anti-alpha-Actinin staining sarcomeric structures, green) and nuclear counterstaining (blue), 30 image fields have been recorded per well and per channel. The entire plate is visualized on the top left. Magnification of these image field (here B12) enables the finding of the EB within each individual well. A larger magnification of these aggregates (bottom) shows areas of mESC-derived cardiomyocytes and sarcomeric structures growing out of the EB. ~~To these areas, serial script-based filtering steps can be applied to select the cell population of interest, identify the nuclei and identify of the cytoplasm.~~ **B**) Based on DNA staining (Hoechst 33342), the nuclear area was summed up for all 30 image fields per well. If no EB is transferred into the image plate during the automation process (as verified by visual inspection) wells do not contain nuclear staining (cut-off criterion mean signal intensity minus three standard deviations) and could systematically be excluded from further analysis (black dots). However, for strongly toxic compounds (hydroxyurea, right panel), a differentiation between a well regarded as “EB not transferred” (black dots) and “EB size strongly affected by compound toxicity” (grey dots, plate layout shown in detail in Figure 5) could not be achieved with this analysis.

**Table S1: Results of the manual murine EST assay. Related to Figure 2.**

|  | **Cytotoxicity assay** | | | | | | **Differentiation assay in hanging drop (petri dish)** | **Compound classification ^1^** |
| --- | --- | --- | --- | --- | --- | --- | --- | --- |
|  | **MTT, 7 d** | | **CTG, 7 d** | | **CTG, 5 d** | |  |  |
|  | IC_50_ (µg/ml) mESC-D3 | IC_50_ (µg/ml) NIH/3T3 | IC_50_ (µg/ml) mESC-D3 | IC_50_ (µg/ml) NIH/ 3T3 | IC_50_ (µg/ml) mESC-D3 | IC_50_ (µg/ml) NIH/3T3 | ID_50_ (µg/ml) mESC-D3 |  |
| **Saccharin** | > 1000 (> 1000; > 1000)  (ZEBET 3498) | > 1000 (> 1000; > 1000)  (ZEBET 3000) | > 1000 (> 1000; > 1000) | > 1000 (> 1000; > 1000) | 464 (464; > 1000) | > 1000 (> 1000; > 1000) | > 1000 (> 1000; > 1000)  (ZEBET 2000) | no  embryotoxicity |
| **Penicillin G** | > 1000 (>1000; > 1000)  (ZEBET 2950) | > 1000 (> 1000; > 1000)  (ZEBET 1586) | > 1000 (> 1000; > 1000) | > 1000 (> 1000; > 1000) | > 1000 (> 1000; > 1000) | 992 (> 1000; 992) | > 1000 (> 1000; > 1000)  (ZEBET 3450) | no  embryotoxicity |
| **Caffeine** | 152 (> 200; 152)  (ZEBET 165) | 145 (145; > 200)  (ZEBET 155) | 119 (119; > 200) | > 200 (> 200; > 200) | 172 (146; 199) | > 200 (> 200; > 200) | 130 (130; > 200)  (ZEBET 185) | weak  embryotoxicity |
| **Dexamethasone** | 23.5 (26.1; 20.9)  (ZEBET 23) | 49.2 (49.2; > 50)  (ZEBET 26) | 20.8 (20.3; 21.3) | 35.2 (35.2; < 50) | 15.7 (17.4; 14.0) | 34.8 (32.5; 37.1) | 25.7 (29.1; 22.3)  (ZEBET 18.3) | weak  embryotoxicity |
| **5-Fluoruracil** | 0.0395 (0.0444; 0.0346)  (ZEBET 0.103) | 0.116 (0.115; 0.117)  (ZEBET 0.17) | 0.0681 (0.0658; 0.0705) | 0.128 (0.123; 0.132) | 0.0493 (0.0485; 0.0501) | 0.0958 (0.0997; 0.0918) | 0.0457 (0.0458; 0.0436)  (ZEBET 0.0289) | strong  embryotoxicity |
| **Hydroxyurea** | 2.11 (2.08; 2.15)  (ZEBET 2.0) | 2.17 (1.81; 2.53)  (ZEBET 7.2) | 2.37 (2.28; 2.46) | 4.83 (> 10; 4.83) | 3.19 (4.12; 2.26) | 3.12 (> 10; 3.12) | 2.55 (2.56; 2.54)  (ZEBET 1.7) | strong  embryotoxicity |

**Table S2: Results of the manual human EST assay. Related to Figure S1.**

|  | **Cytotoxicity assay** | | | |
| --- | --- | --- | --- | --- |
|  | **MTT, 5 d** | | **CTG, 5 d** | |
|  | IC_50_ (µg/ml) ZIPi013-E | IC_50_ (µg/ml) HDFf | IC_50_ (µg/ml) ZIPi013-E | IC_50_ (µg/ml) HDFf |
| **Penicillin G** | 749.9 | 821.5 | > 1000 | 794.6 |
| **Caffeine** | 13.27 | 358.5 | 43.63 | 411.8 |
| **Hydroxyurea** | 0.3326 | 4.005 | 0.8652 | 30.15 |
| **5-Fluorouracil** | 0.2234 | 0.1953 | 0.0166 | 0.1864 |

**Supplementary Experimental Procedures**

**High content imaging of EB outgrowths**

After transfer of the EBs to the imaging plates, mESCs were fixed with 4 % paraformaldehyde in PBS for 10 minutes at ambient temperature. After two washes with cold PBS, cells were permeabilized with 0.1 % Triton X-100 (Roth, Cat. No. 3051) in PBS for 15 minutes followed by a single washing step with PBS. For staining, cells were blocked with 5 % FCS in PBS for 30 minutes. Cells were incubated with cardiomyocyte-specific antibodies diluted 1:200 in blocking solution (anti-Sarcomeric Alpha Actinin (rabbit monoclonal) (abcam, Cat. No. ab68167, Lot. No. GR239387-7), anti-Myosin Heavy Chain (mouse monoclonal IgG2B) (R&D, Cat. No. MAB4470, Lot. No. CAEI0314091)) over night at 4° C and primary antibodies were detected with secondary antibodies diluted 1:500 in blocking solution (Alexa Fluor 488 donkey anti-rabbit IgG (Invitrogen, Cat. No. A21206, Lot. No. 1028736), Alexa Fluor 647 donkey anti-mouse IgG (Invitrogen, Cat. No. A31571, Lot. No. 940076)) for 1 h at ambient temperature in the dark. Nuclear counterstaining was performed with 1 µg/ml Hoechst 33342 (Invitrogen, Cat. No. H3570) in PBS for 20 min. Image acquisition and analysis was carried out on an Opera High Content Imaging System in combination with the Columbus Image Data Storage and Analysis System (PerkinElmer). Laser power and exposure times were adjusted to a linear detection range. Automated imaging was performed using a 20x water immersion objective and a sub layout with 30 evenly distributed and not overlapping image fields per well of a 96-well imaging microplate. Based on DNA staining (Hoechst 33342), the nuclear area was summed up for all 30 image fields per well.

**Supplementary References**

1. Seiler, A.E. & Spielmann, H. The validated embryonic stem cell test to predict embryotoxicity in vitro. *Nature protocols* **6**, 961-978 (2011).
